# Study of biopolymer encapsulated Eu doped Fe_3_O_4_ nanoparticles for magnetic hyperthermia application

**DOI:** 10.1038/s41598-024-60040-7

**Published:** 2024-04-29

**Authors:** Krishna Priya Hazarika, J. P. Borah

**Affiliations:** Nanomagnetism Group, Department of Physics, National Institute of Technology Nagaland, Dimapur, Nagaland 797103 India

**Keywords:** Magnetic hyperthermia, Dipolar interactions, Magnetic anisotropy, Saturation magnetization, Biopolymers, Materials science, Nanoscience and technology

## Abstract

An exciting prospect in the field of magnetic fluid hyperthermia (MFH) has been the integration of noble rare earth elements (Eu) with biopolymers (chitosan/dextran) that have optimum structures to tune specific effects on magnetic nanoparticles (NPs). However, the heating efficiency of MNPs is primarily influenced by their magnetization, size distribution, magnetic anisotropy, dipolar interaction, amplitude, and frequency of the applied field, the MNPs with high heating efficiency are still challenging. In this study, a comprehensive experimental analysis has been conducted on single-domain magnetic nanoparticles (SDMNPs) for evaluating effective anisotropy, assessing the impact of particle-intrinsic factors and experimental conditions on self-heating efficiency in both noninteracting and interacting systems, with a particular focus on the dipolar interaction effect. The study successfully reconciles conflicting findings on the interaction effects in the agglomeration and less agglomerated arrangements for MFH applications. The results suggest that effective control of dipolar interactions can be achieved by encapsulating Chitosan/Dextran in the synthesized MNPs. The lower dipolar interactions successfully tune the self-heating efficiency and hold promise as potential candidates for MFH applications.

## Introduction

In light of the emerging biomedical applications enabled by magnetic nanoparticles (MNPs), such as bioimaging, drug delivery, biosensors, and magnetic fluid hyperthermia (MFH), understanding and managing particle-intrinsic characteristics, colloidal parameters, and spatial coordination play a crucial role in determining the MNPs magnetic relaxation behavior^1^. As a result, these parameters significantly influence MFH as a cancer cure, as they determine the energy losses converted into heat from colloidal magnetic nanoparticles under a high-frequency AC magnetic field^2^. Presently, the optimization of magnetic nanoparticle (MNP) heating efficiency is centered around designing MNPs with tailored properties, such as specific size, effective magnetic anisotropy (*K*_*eff*_), or saturation magnetization (*M*_*s*_). Nevertheless, the challenge of simultaneously optimizing all characteristics under diverse experimental characteristics for magnetic hyperthermia remains unresolved. Recent studies have demonstrated that interparticle interactions have notable effects on the relaxation time, blocking temperature, and the hysteresis loops or specific absorption rate (SAR), with additional analyses focused on heat dissipation within agglomerated systems^3^. Despite numerous studies and diverse approaches on the topic, the impact of dipolar interactions on the SAR of suitably designed MNP systems remains a subject of ongoing controversy. Among the MNPs, spinel ferrite Fe_3_O_4_ has encountered much insight into biomedical applications due to its high biocompatibility and suitable magnetic properties^1,2^. Therefore, the advancement of nanomaterials, and one notable example is stabilizing the Fe_3_O_4_ (FO) nanoparticles via different dopants in the FO matrix. Following the literature, RE ions doped in an octahedral site of the Fe_3_O_4_ system can stabilize and reduce the conversion possibility to maghemite or hematite^3,4^. Addedly, Eu-doped spinel ferrite MNPs draw significant attention, due to their biocompatibility and their unique magnetic and optical properties^1^. In literature, engineered Eu-doped nanoparticles provoke interest in multimodal therapeutic applications owing to their enhanced magnetic resonance imaging (MRI) properties and the nature of biocompatibility^1,2^. Furthermore, because of the analogous ionic radii of Fe and Eu, the lesser doping concentration would not lead to the variation of the structural configuration, which pays a great deal of prominence to the researchers. This study aims to extend our research on the Eu doping of Fe_3_O_4_ MNPs by introducing a coating agent (chitosan/dextran) and investigating the role of dipolar interactions and effective magnetic anisotropy in enhancing heating performance^4^. In the previous research, Eu-doped Fe_3_O_4_ (EuFO) matrix with different concentrations has been studied, with a primary focus on investigating the role of site-preferred substitution to tune magnetic and structural parameters to improve the self-heating efficiency for MFH applications. In this context, 7% Eu doped FO MNPs show an optimum result for MFH applications, and the study highlights that the dispersion of MNPs in aqueous solutions poses an additional challenge, primarily due to their mutual magnetic interactions and high surface energies, leading to the tendency of MNPs to agglomerate. The clustered MNPs often promote MNPs-density inhomogeneities, producing non-homogenous heating in the synthesized samples. To counter this consequence on MFH performance, the present consensus is that the engineered MNPs should possess controlled aggregation to consider the dominant contribution of dipolar interactions to correctly determine the magnetic characteristic parameters. Furthermore, based on the existing literature, the widely adopted approach to control aggregation is the effective conjugation of biomolecules such as peptides, oligonucleotides, antibodies, and natural polymers^1,7–11^. According to previous studies, chitosan and dextran have emerged as highly promising candidates for encapsulating engineered RE-doped FO MNPs due to their non-antigenic, biocompatible, bio-functional, and biodegradable nature^10–19^. In this work, a novel experimental approach is presented to accurately understand the role of dipolar interactions and heating efficiency, with the specific goal of distinguishing energy contributions towards MFH applications.

## Experimental sections

### Synthesis of the MNPs

**Figure 1:**
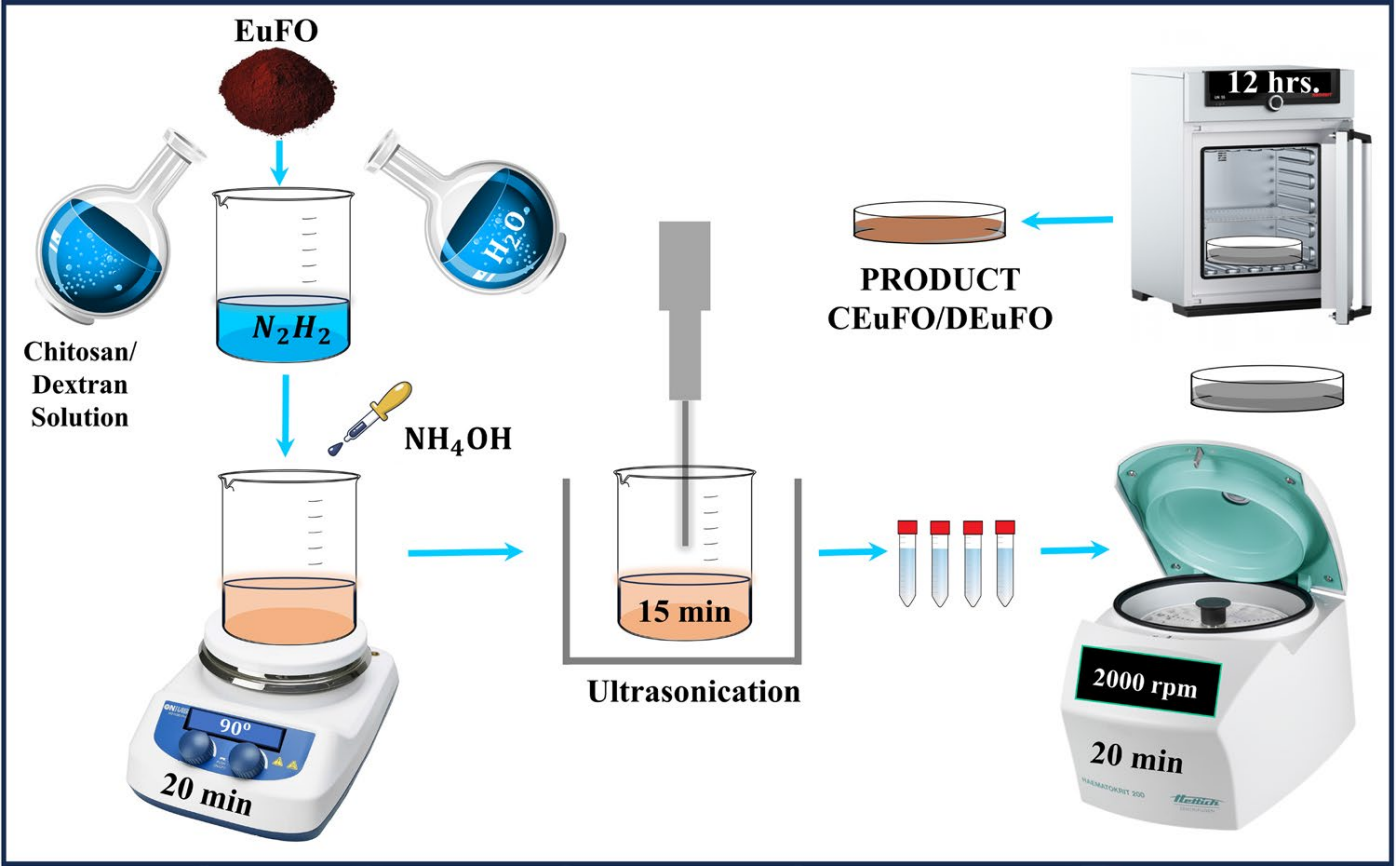
Schematic representation of the synthesized MNPs.

The synthesizing technique for EuFO MNPs is explained thoroughly in the prior work^4^. The synthesis of Eu-doped Fe_3_O_4_ MNPs coated with chitosan and dextran has followed the identical procedure outlined in the prior study^18^. Henceforth, only pen the brief expression of the synthesizing route for chitosan and dextran-coated Eu-doped Fe_3_O_4_ MNPs and it is comprehended in Fig. 1.

All the reference MNPs were synthesized using the co-precipitation method, and the resulting EuFO was additionally surface-modified with biopolymers, specifically chitosan and dextran, referred to as CEuFO and DEuFO in this article. In the experimental protocol, 0.25 g of chitosan was dispersed in 100 ml of a 0.1 M acetic acid solution, and this mixture was slowly added drop by drop to 0.5 g of EuFO suspended in 10 ml of milli-Q water. The stirring was maintained at 180 rpm for 20 min, followed by a 15-min ultrasonication period. Following this, the prepared MNPs were washed with a mixture of ethanol and double-deionized water, then subjected to centrifugation at 2000 rpm for 20 min. The resultant precipitate obtained from the experiments underwent drying in a vacuum oven and was eventually pulverized into a powder. The procedure was extended for the development of DEuFO MNPs and in this instance, dextran was dispersed directly in double-deionized water instead of a 0.1 M acetic acid solution.

### Characterization

The phase purity and crystallographic details of the designed magnetic nanoparticles were analysed using X-ray diffraction (Rigaku Ultima IV), and the XRD findings were directed with Cu-Kα radiation (λ = 1.5406 Å). The functional groups and elemental states were studied using Fourier transform infrared spectra (FTIR) analysis accomplished on an Agilent Technology Cary 630 instrument. The morphology and average particle size were premeditated via ZEISS Gemini 300 field-emission scanning electron microscope (FESEM), while the selected area electron diffraction (SAED) and d-spacing measurements were investigated with JEOL, JEM 2100 high-resolution transmission electron microscope (TEM). The electronic and chemical states of the MNPs were widely observed using X-ray photoelectron spectroscopy on a Thermo Fisher Scientific Excalab Xi + instrument. X-ray radiation from an Al Kα source was employed for the analysis. The magnetic parameters of the nanoparticles were examined by a Lakeshore 7410 series vibrating sample magnetometer (VSM) and Electron Spin Resonance (ESR; JEOL, JES-FA200) techniques. The processed MNPs underwent a self-heating study via induction heating setup (Easy Heat-8310, Ambrell make, U.K.).

## Results and discussion

### Structure and morphology study

**Figure 2:**
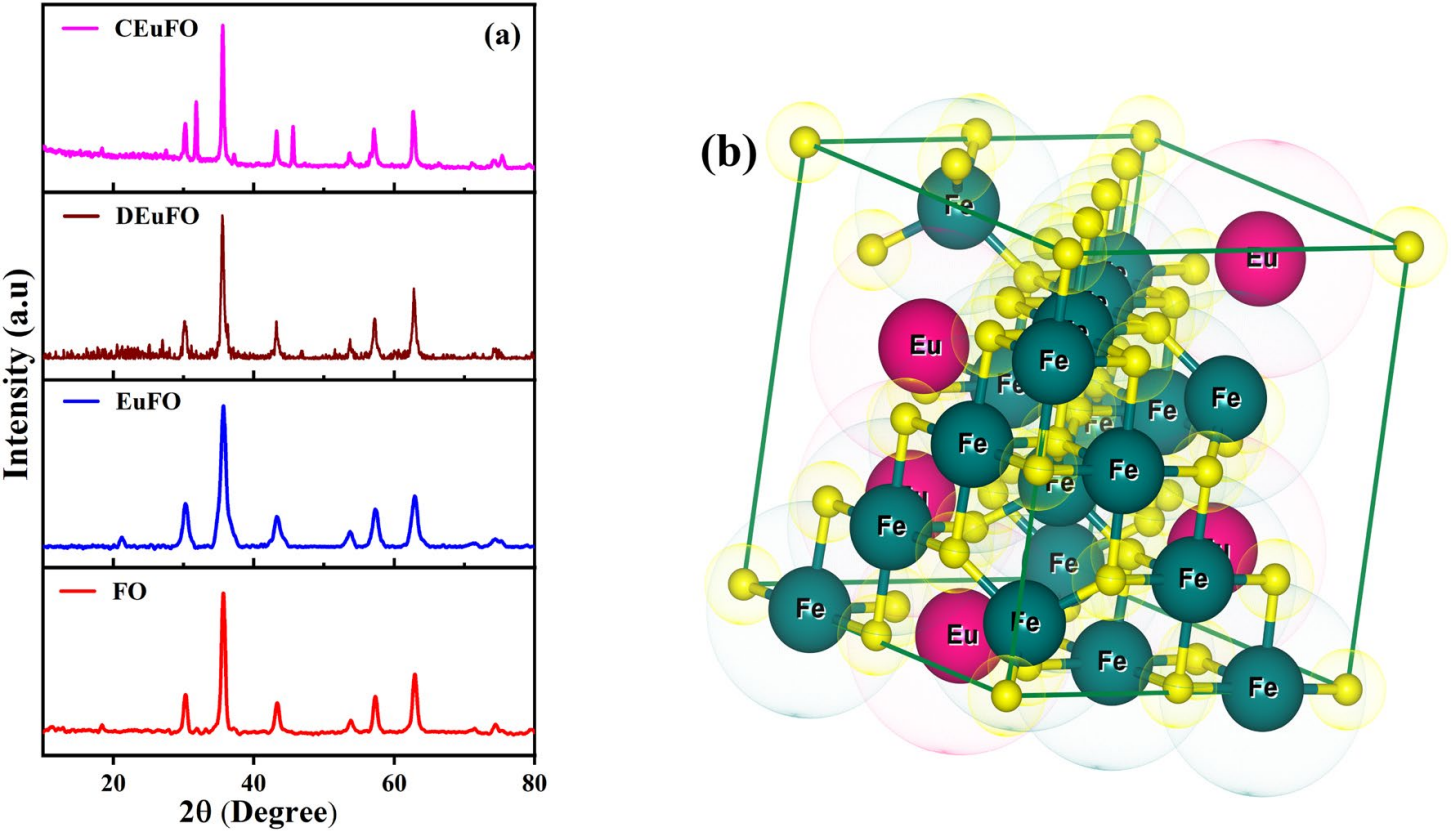
(**a**) XRD; Rietveld refinement of the reference MNPs (**b**) Ball-and-stick model of *fcc* cubic spinel structure of EuFO MNPs.

The XRD spectra of engineered dextran and chitosan-functionalized Eu doped FO MNPs, as displayed in Fig. 2, portray a pure crystalline phase, which exhibits distinct diffraction peaks ascertained as (220), (311), (400), (511), and (440). Herein, the observed peaks in the X-ray diffraction pattern can be attributed to the cubic spinel structure of Fe_3_O_4_, which aligns with the data documented in the ICDD PDF card number 01–075-0033 with space group *Fd* $\overline{3 }$ *m*^19^. Furthermore, due to the non-crystalline structure of dextran and chitosan, the final product matrix (DEuFO/CEuFO) indicates no alteration of the crystal structure^20^. Figure 2b gives a precise schematic representation of Eu doped ferrite (generated from VESTA software), which resembles that Eu ions choose to be in the B sites rather than A sites.

The computed structure parameters are enclosed in Table 1, where crystallite size was measured by using the Scherrer formula^26^. Remarkably, the increasing trend of crystallite size and cell volume indicate the influence of chitosan and dextran. Moreover, the rising trend in crystallite size aligns with the support of TEM analysis. The polymers may affect nucleation and growth processes, leading to larger crystallites. The observed changes in the crystal structure attributed to developed lattice strain^4^, stress, cation re-arrangement, and finite size effect^27^ can be attributed to the active involvement of chitosan and dextran, which bring about structural modifications at the atomic and molecular levels. To counter this effect, the variation in dislocation density with the encapsulation of chitosan and dextran highlights the influence of the densification progression, which is sensible due to the physical characteristics of cations, their valence states, and distribution on A and B sites.

**Table 1:** Crystallite size, particle size, strain (ε), cell volume, and dislocation density (U+12E8_D_).

Sample name	FO	EuFO	CEuFO	DEuFO
Crystallite size (nm)	14.21	15.57	16.71	16.79
Particle size D_TEM_ (nm)	14 ± 3	15 ± 3	17 ± 2	17 ± 3
ε	0.004	0.005	0.006	0.005
Cell volume (Å)	581.65	582.29	582.50	582.52
U+12E8_D_ (× 10^–2^)	0.39	0.38	0.38	0.36

**Figure 3:**
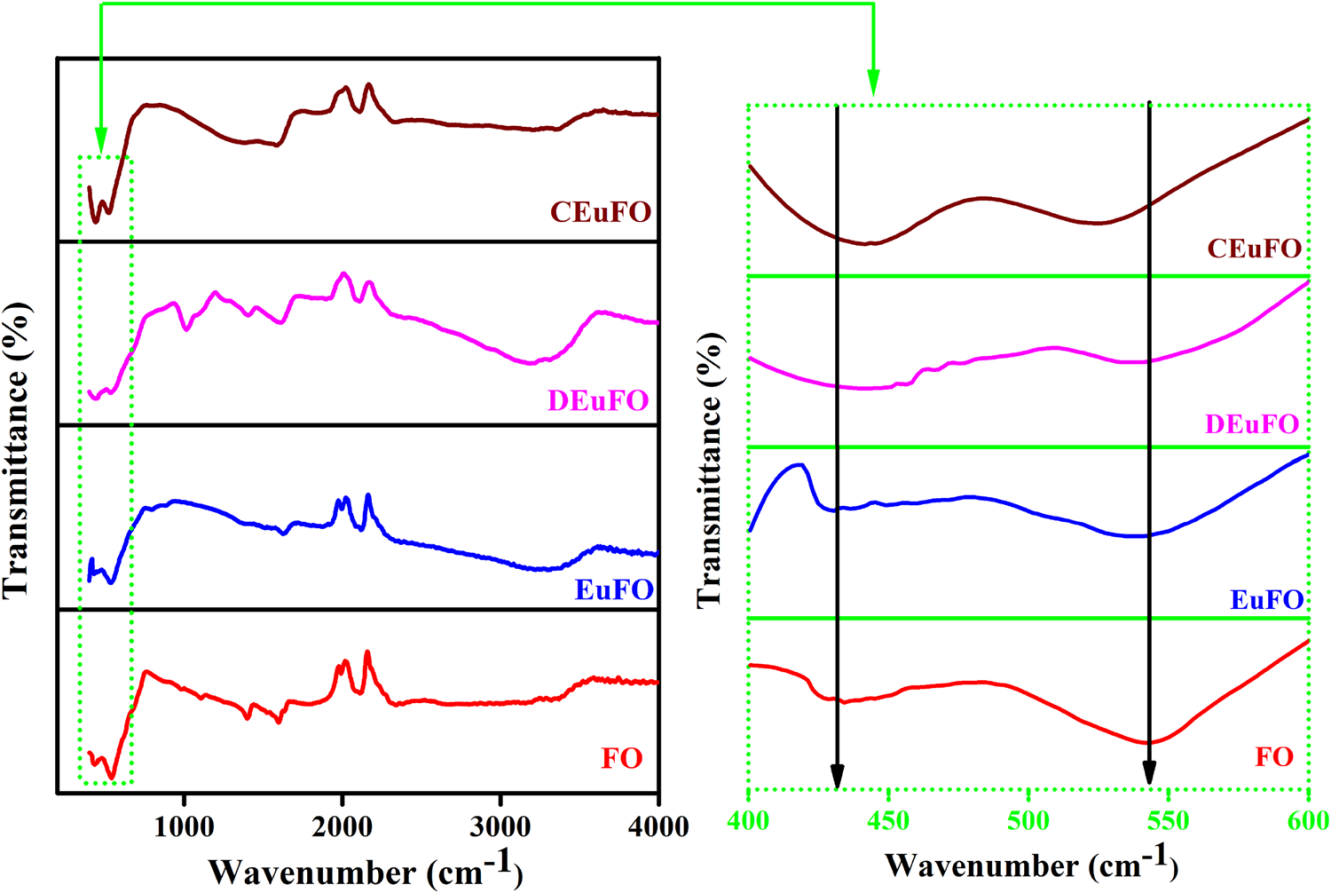
FTIR spectra of the synthesized MNPs.

Figure 3 illustrates the FTIR spectra of the engineered MNPs, under the frequency limit 400–4000 cm^−1^. In this regard, a broad absorption band of approximately ~ 3450 to 3550 cm^−1^ was observed, corresponding to the stretching vibrations of O–H bonds in the absorbed H_2_O molecule, and this observation aligns with the documented previous literature^31^. To continue with this, the presence of two extra absorption peaks at approximately 1595 cm^−1^ and 1371 cm^-1^ suggests the signature of N–H bending and C–O stretching, strongly signifying the successful functionalization of chitosan on the surface of the processed CEuFO MNPs^30,31^. Consequently, the DTbFO spectra exhibit an extra peak in the limit of nearly 1000–1250 cm^−1^, which can be ascribed to the signature C–O and C–O–C stretching of the polymeric chain of dextran, indicating the effective encapsulation of dextran in the respective EuFO system^8^. However, the processed MNPs display distinct absorption peaks at approximately 428 and 540 cm^-1^ in their spectrum, indicating signature traits of the spinel ferrite structure^33^. More in detail, the absorption peaks at around 540 cm^−1^ (υ_1_) and 428 cm^−1^ (υ_2_) are a result of the stretching vibration of the Fe–O bonds in the tetrahedral (A) and octahedral (B) metal complexes, respectively^34^. Furthermore, Fig. 3 demonstrates the shift of the absorption band (υ_2_) towards higher wavenumbers at approximately 428 cm^−1^ due to the presence of Europium ions in the octahedral sites of the FO matrix, which aligns with the findings obtained from the computed XRD analysis.

**Figure 4:**
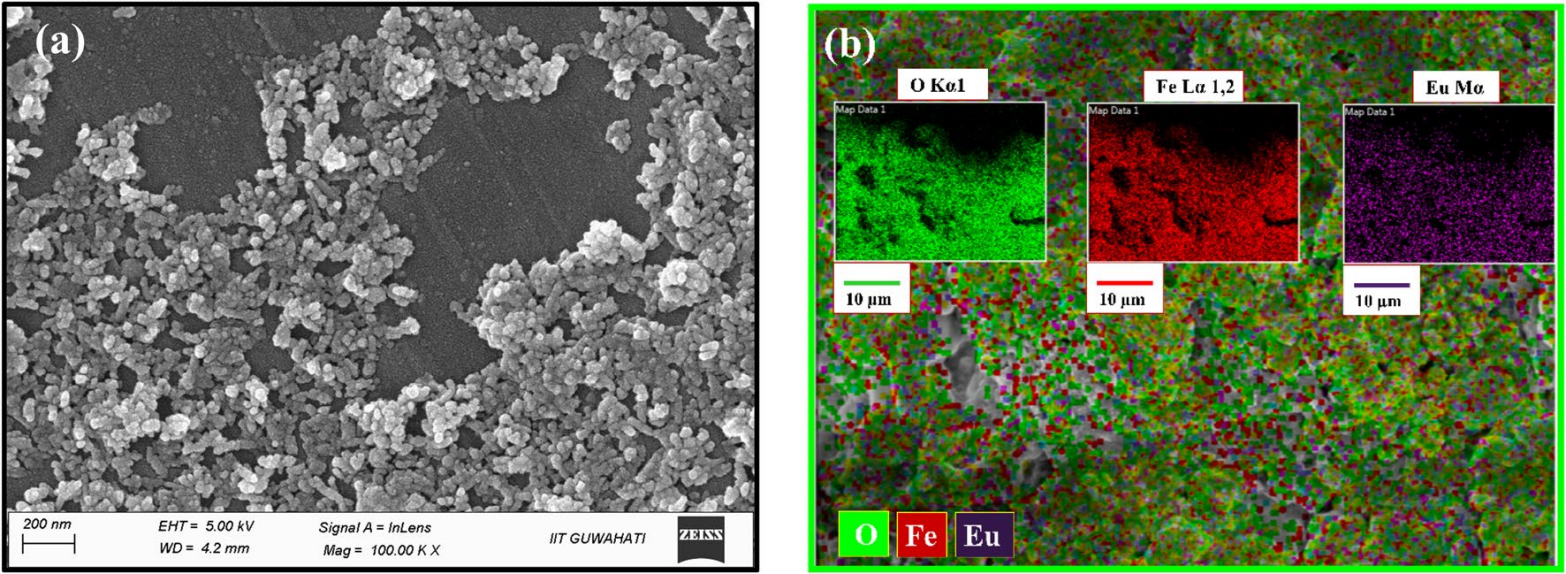
(**a**) SEM image of CEuFO MNPs (**b**) Elemental mapping of EuFO MNPs.

The surface morphology of the reference MNPs is studied via SEM analysis, where Fig. 4a portrays the chitosan-coated Eu-doped FO MNPs. The SEM micrograph of CEuFO MNPs shows a nearly uniform, berry-like structure. In addition, elemental mapping from SEM images (EuFO MNPs) conveys the existence of the elements Fe, O, and Eu, respectively, as depicted in Fig. 4b.

**Figure 5:**
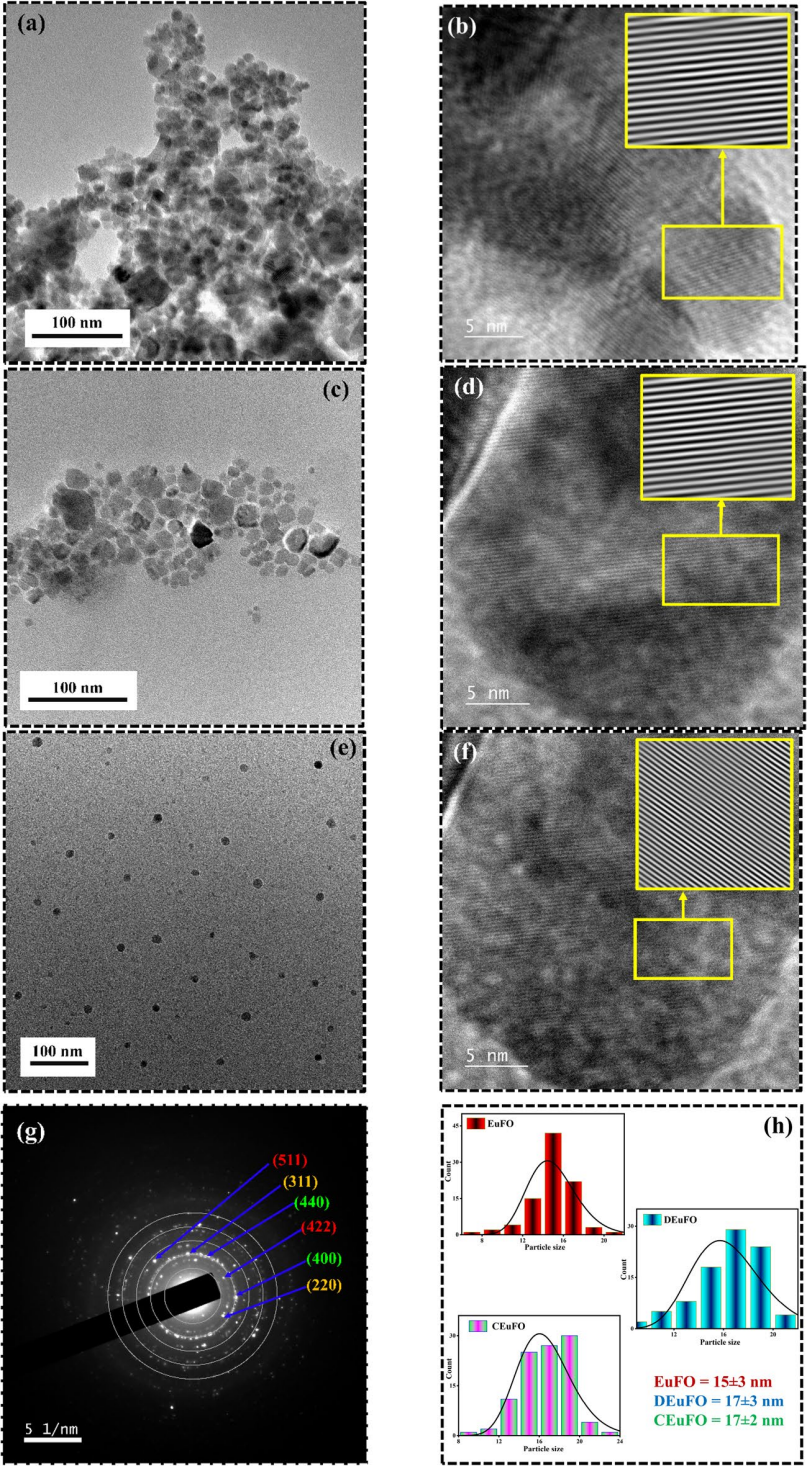
TEM images: (**a**) EuFO MNPs (**b**) d-spacing of EuFO MNPs (**c**) DEuFO MNPs (**d**) d-spacing of DEuFO MNPs (**e**) CEuFO MNPs (**f**) d-spacing of CEuFO MNPs (**g**) SAED inset of DEuFO MNPs (**h**) lognormal distribution for all the reference MNPs.

The TEM images of the reference MNPs are portrayed in Fig. 5. The effective addressing of excellent dispersion is demonstrated through the TEM images, indicating that the designed synthesis approach efficiently prevents agglomeration issues. These findings are in excellent agreement with the observations obtained from SEM analysis. Additionally, the d-spacing values consistent with the (311) plane of the characterized EuFO, CEuFO, and DEuFO MNPs match perfectly with the calculated XRD data (Fig. 5b,d,f). The particle size is calculated through the lognormal distribution (Fig. 5h), and the estimation method is strongly aligned with the XRD findings, as depicted in Table 1. The increment in particle size can be accredited to the co-existence of polymers (chitosan/dextran) and dopants (Eu), resulting in particle size and shape modification. The difference in nanoparticle size between chitosan and dextran coatings could be attributed to charges from chitosan macromolecules, which are absent in dextran-coated MNPs. Conversely, the positively charged amino groups in chitosan-coated MNPs create electrostatic repulsion among the CEuFO, preventing their aggregation and forming smaller, isolated particles^17^. In this regard, the absence of charges (amino group) in dextran can contribute to partial agglomeration of the MNPs, foremost to an increase in particle sizes. The presence of concentric circles with bright spots in the SAED pattern is depicted in Fig. 5g, signifying that the MNPs are polycrystalline in nature^35^. The spotty rings observed in the SAED pattern are consistent with the Bragg planes and align well with the results obtained from XRD analysis.

**Figure 6:**
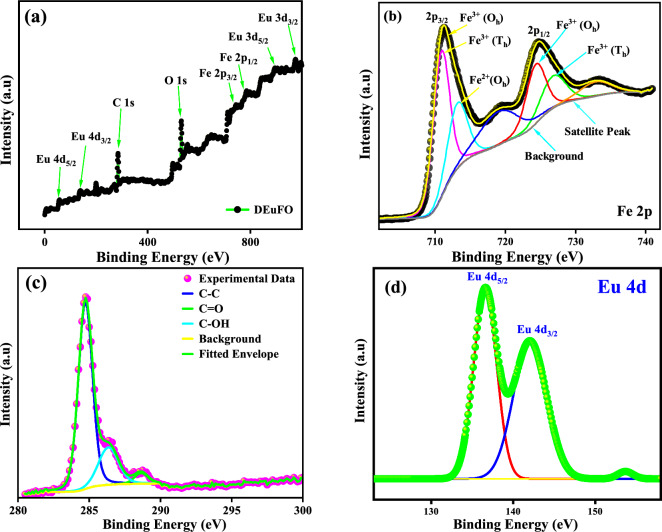
XPS scans of DEuFO; (**a**) complete survey of DEuFO MNPs (**b**) Fe 2p spectra (**c**) C 1 s spectra (**d**) Eu 4d spectra.

The effective doping and coating of the processed MNPs can be investigated via XPS analysis as enclosed in Fig. 6. The overall survey scans of DEuFO MNPs are displayed in Fig. 6a, implying the successful functionalization of dextran (composed of hydroxyl group) and proper doping of rare-earth element (Eu) in the FO matrix. Following the scan results in Fig. 6b, the satellite Fe peaks examined in the scan correspond to the formation of bonds initiated by both octahedral (O_h_) and tetrahedral sites (T_h_), exhibiting valence states of + 2 and + 3. The high-resolution core Fe 2p electron spectra of DEuFO MNPs show distinct peaks at approximately ~ 711 eV and ~ 722 eV, corresponding to the spin-orbital properties of 2p_3/2_ and 2p_1/2_, respectively^36^. The satellite peak of the C 1 s scan in Fig. 6c provides a deep insight into the existence of dextran in the system. The deconvolution of Eu 4d_5/2_ and Eu 4d_3/2_, revealing approximate binding energies of around ~ 136 eV and ~ 143 eV (Fig. 6d), indicates the presence of two bonds initiating from the Eu ions.

### Magnetic analysis

**Figure 7:**
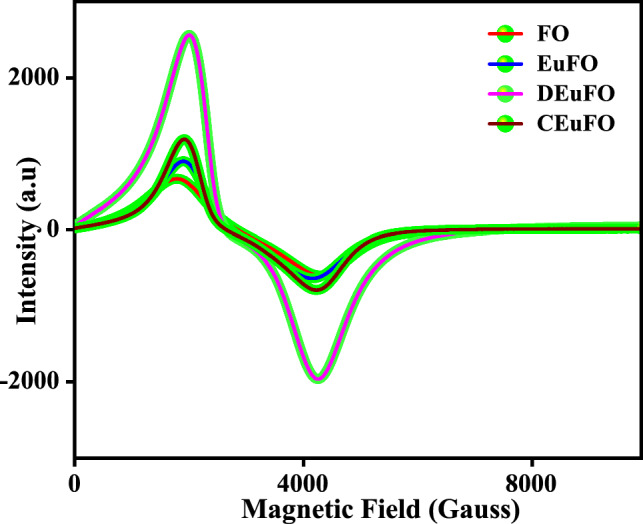
ESR spectra of the reference MNPs.

The magnetic characteristics and spin-related phenomena of the magnetic nanoparticles (MNPs) were assessed using ESR spectroscopy, as depicted in Fig. 7. The observed broadening in the ESR spectra reveals a Lande g factor near to ~ 2, providing evidence for the presence of a superparamagnetic phase in the synthesized magnetic nanoparticles (MNPs)^37^. In particular, the observed broadening of the curves signifies the predominance of dipolar interactions^38^. The results obtained from the ESR findings are presented in Table 2, encompassing essential characteristics such as spin–spin relaxation time (τ_1_), spin–lattice relaxation time (τ_2_), resonance field (H_r_), resonance linewidth (H_pp_), effective g value, and full width at half maximum (FWHM) of the absorption spectra (ΔH_1/2_ = √3 H_pp_)^39^. Herein, the diminished magnitudes of both *g* and H_pp_ values indicate the substantial influence of potent super-exchange interactions. Subsequently, the reduced *g* and H_pp_ values observed in the case of dextran and chitosan-coated MNPs in comparison to bare and Eu-doped MNPs exemplify a random orientation of magnetic moments, concurrent with the VSM study^41^.

**Table 2:** Measured ESR parameter (spin–spin relaxation time τ_1_, spin–lattice relaxation time τ_2_, effective g value, resonance linewidth H_pp_, FWHM of the absorption spectra ΔH_1/2_, and resonance field H_r_).

Sample details	τ_1_ × 10^–11^ (sec)	τ_2_ × 10^–12^ (sec)	$g$	H_pp_ (Gauss)	ΔH_1/2_ (Gauss)	H_r_ (Gauss)
FO	6.85	1.03	2.02	2530	4382	2835
EuFO	7.51	0.94	2.02	2219	3843	2728
DEuFO	7.30	0.97	2.02	2218	3841	2652
CEuFO	7.27	0.98	2.02	2217	3839	2640

Moreover, within the context of the magnetic fluid hyperthermia (MFH) investigation, the SAR emerges as a basic threshold for quantifying the self-heating efficacy of the magnetic assembly, and it relates inversely to the relaxation time. As enclosed in Table 2, the recorded decrease in spin–spin relaxation time (τ_1_) for the coated MNPs compared to the uncoated counterpart signifies a higher SAR, further confirmed by induction heating analysis^39^.

**Figure 8:**
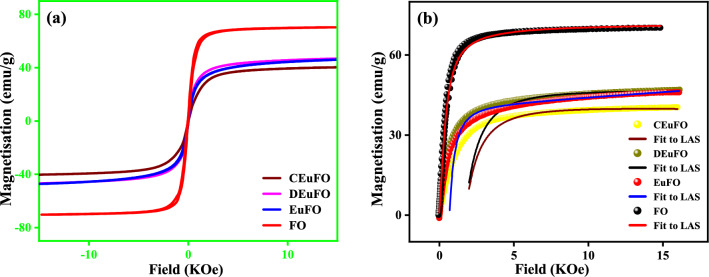
(**a**) M–H hysteresis loop for the reference MNPs (**b**) LAS fit of the reference MNPs.

The magnetic parameters of the synthesized MNPs are represented in Fig. 8. The S-shaped hysteresis graphs were subjected to analysis using the Langevin fit, unveiling the nearly superparamagnetic behavior of the nanoparticles^43^.

For precise assessment of saturation magnetization (*M*_*s*_) and effective anisotropy constant (*K*_*eff*_), employing the Law of Approach to Saturation Magnetization (LAS) to fit the S-shaped graphs^44^. The attained magnetic characteristics, comprising saturation magnetization (*M*_*s*_), effective anisotropy constant (*K*_*eff*_), retentivity (*M*_*r*_), and coercivity (*H*_*c*_), are enclosed in Table 3.

**Table 3:** Magnetic parameters: Saturation magnetization (*M*_*s*_), Coercitivity (*H*_*c*_), Retentivity (*M*_*r*_), and effective anisotropy constant (*K*_*eff*_).

Sample details	*M*_*s*_ (emu/g)	*H*_*c*_ (Oe)	*M*_*r*_ (emu/g)	*k*_*eff*_ × 10^5^ (erg/cm^3^)
FO	70.61	22	2.59	2.56
EuFO	46.26	9	1.21	2.26
DEuFO	46.02	6	0.82	1.79
CEuFO	40.89	4	0.76	2.58

As evidenced by the magnetic findings presented in Table 3, it is observed that the M_s_ of the EuFO MNPs parades a significant decrease compared to the pure FO MNPs. The reduction in M_s_ observed here is primarily attributed to the impact of size effect and spin randomness induced through spin canting at the surfaces^45^, which signify a direct concurrence between the magnetic and structural states of the examined MNPs^46^. As revealed by XRD analysis, the substitution of Eu^3+^ cations into the octahedral Fe^3+^ sites leads to elongation in bond lengths Fe^3+^(Eu^3+^)-O^2-^, and as the bond lengths elongate, the coordination of cations weakens, resulting in a more pronounced oxygen deficiency; notably, this reduces exchange interaction and magnetic parameters^47^. Albeit, the reduction in *M*_*s*_ observed in coated MNPs can primarily be ascribed to surface-induced spin canting, magnetic dilution from the non-magnetic characteristics of chitosan and dextran coatings, along with interfacial effects and collective oscillations^48^. Additionally, the presence of a coating on the samples inhibits aggregation that arises due to dipolar interactions among magnetic cores, consequently enhancing the colloidal stability. This characteristic also brings the advantage of restraining the interaction between Fe^2+^ ions and enzymes, subsequently lowering the promotion of reactive oxygen species generation through the Fenton reaction^50^. Besides, the Rietveld refinement further corroborates the reconfiguration of cation distribution within the CEuFO and DEuFO MNPs. The observed increase in the *M*_*s*_ of DEuFO comparison to CEuFO can be accredited to the ligand characteristics of chitosan and dextran, where ligands act to influence the crystal field splitting following the principles of ligand field theory^51,52^. The findings suggest that dextran-coated MNPs prefer a high spin state of Fe^3+^ within the octahedral site, owing to the presence of a hydroxyl group serving as a weak field ligand^53^. Conversely, chitosan-coated MNPs display a greater preference for the low spin state of Fe^3+^ within the octahedral site, primarily attributed to the prevalence of the amine group in chitosan^51^. According to the linear response theory, magnetic anisotropy plays a vital role in controlling the heating efficiency of the nanoparticles^54^. In this regard, diverse approaches on the topic, the impact of effective anisotropy^55^ on the SAR of suitably designed MNP systems remains a subject of ongoing controversy. Moreover, theoretically Fu et al.^56^ and experimentally Serantes et al.^57^ highlighted that these chain-like structures positively impact the magnetic anisotropy and lead to the enhancement of the heating efficiency. Luis et al. investigated the nanoparticle heating efficiency and reported that the heating efficiency has profoundly amplified with low anisotropy^58^. Consistent with the literature, particularly in the magnetic assessment detailed in Table 3 highlights a reduction of effective magnetic anisotropy (*K*_*eff*_) within the dextran and chitosan-coated MNPs compared to the uncoated FO and EuFO MNPs. In this context, the decrease in effective magnetic anisotropy (*K*_*eff*_) observed in DEuFO and CEuFO MNPs can be linked to a weakened interaction between Eu and Fe, resulting in a lowered ratio of spin orbital moments of 4f. electrons and subsequent diminution of the spin–orbit couplings^59^. In addition, from the Stoner-Wohlfarth model^60^, it is evident that the reducing trend in coercivity observed in the reference nanoparticles results from a direct correlation with the reduced value of the effective anisotropy constant^60^.

### Self-heating efficiency study

**Figure 9:**
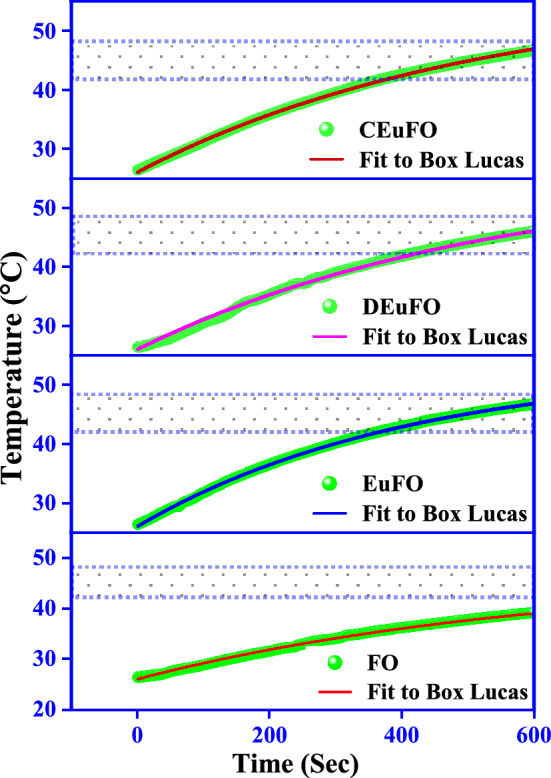
Box-Lucas fit of time-dependent temperature deviation curve for synthesized MNPs for 1 mg/mL concentrations.

The magnetic heating efficiency of the reference MNPs for magnetic hyperthermia applications is expected to be triggered by an AC magnetic field at a frequency of 337 kHz, with an amplitude of 249 A and a magnetic field strength of 14.92 kAm^−1^, under the maintenance of clinical safety thresholds (H.*f* ≤ 5 × 10^9^ Am^−1^ s^−1^)^61^. Figure 9 illustrates the time-dependent temperature profile at a concentration of 1 mg/mL, wherein the Box-Lucas model^62^ was applied to determine the specific absorption rates (SAR) and intrinsic loss power (ILP) of the reference MNPs. In particular, maintaining the hyperthermic range and enhancing the SAR depends on the presence of optimal concentrations of nanoparticles. Theoretically, an optimal concentration for achieving maximum SAR was initially proposed by Haase et al.^63^. and experimentally confirmed by Lahiri et al.^64^. Similarly, Tan et al. elucidated the significant influence of optimal concentration on the heating power dissipation using Monte Carlo simulations^65^. Based on these prior investigations, optimized the concentration to 1 mg/mL, and the graph (Fig. 9) illustrates that all the synthesized nanoparticles are evident in the hyperthermic threshold range (42 °C to 47 °C)^66^. Only a limited number of studies have precisely grasped the influence of particle size distribution on the heating efficiency of MNPs. In this regard, an essential remark was found by Zubarev et al. that the intrinsic interaction of MNPs with diameters of 17–20 nm can significantly lead to up to 30% higher heat production^67^.

**Figure 10:**
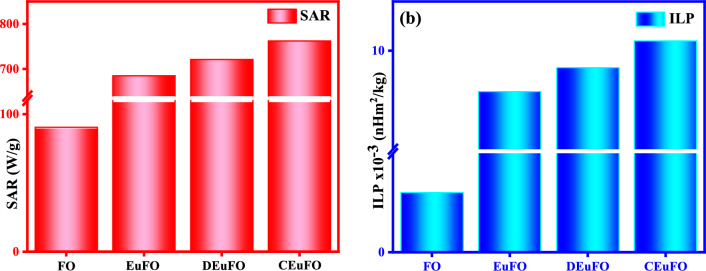
(**a**) SAR Variation (**b**) ILP Variation of the reference MNPs.

**Figure 11:**
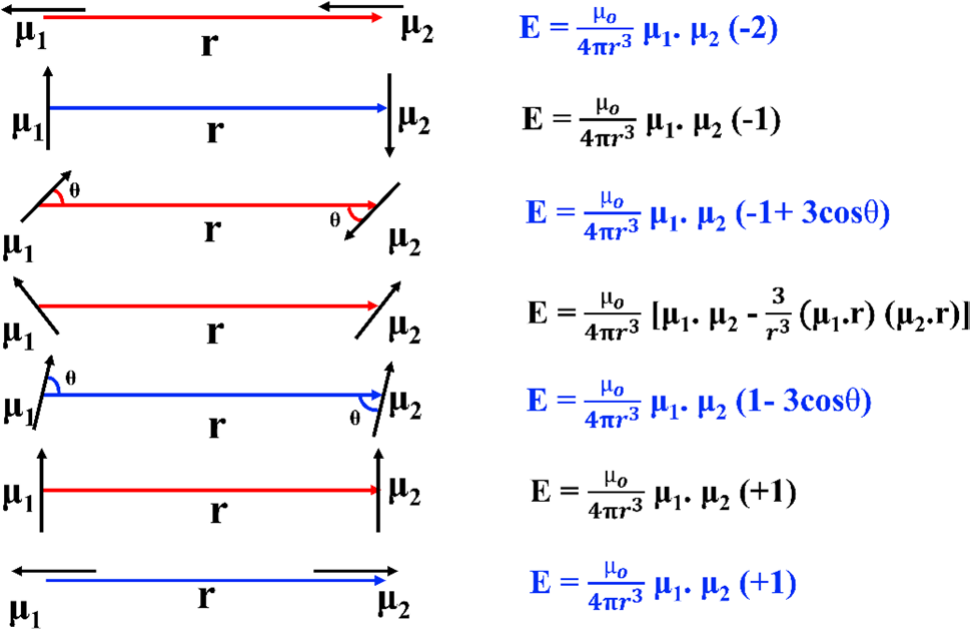
Different alignments for the pair of magnetic dipoles.

In practical scenarios, modifying essential parameters that control magnetic hyperthermia performance, like adjusting the viscosity in particular biological systems or addressing the limitations of nanoparticle rotation within cells or tissues, is challenging^68,69^. Hence, to amplify hyperthermia performance, it is essential to optimize Néel relaxation parameters, which remain unaltered by the biological microenvironment, given the negligible contribution of Brownian relaxation^70^. Analogously, Fig. 10 depicts the variation of SAR and ILP of the reference nanoparticles, and it is observed that even though the magnetic parameters (*M*_*s*_ and *K*_*eff*_) of DEuFO and CEuFO MNPs are lowered, they exhibit improved heating efficiency compared to the uncoated nanoparticles. Nevertheless, the challenge of simultaneously optimizing all parameters under diverse experimental conditions for magnetic hyperthermia remains unresolved. Therefore, it can be noted that another essential parameter is dipolar interaction and it happens to be dominant among all other inter-particle interactions^1^. The dipolar interaction energy in magnetic nanoparticles is directly proportional to 1/r^6^, (r is the inter-particle distance between the particles) indicating that the interaction intensifies as the inter-particle distance decreases. The impact of dipolar interaction on the magnetic relaxations of MNPs, influencing heating efficiency, is subject to debate due to both increasing and decreasing dipolar interactions for self-heating efficiency. To understand the behavior of dipolar interaction-dependent specific absorption rate, Raja Das et al. studied two Fe_3_O_4_ systems as nanospheres and nanocubes^71^. Their results concluded that the weaker intraparticle interactions are preferred as heating mediators. To counter this consequence on MFH performance, Landi et al. reported that dipolar interactions serve to elevate the energy barrier, thereby enhancing the SAR for the magnetically soft particles^72^. Henceforward, the literature reviewed above collectively suggests that the question of achieving optimal heating efficiency remains uncertain, with the roles of magnetocrystalline anisotropy and dipolar interaction. By considering theoretical and experimental findings on heat dissipation via relaxation mechanisms, Dormann-Bessais-Fiorani proposes a modified model for Neel relaxation [τ_N_ = *τ*_0_ exp $(\frac{\Delta E*}{KT})$] in the presence of other energy terms^73^, where ΔE* = E_A_ + E_H_ + E_D_; E_A_ is the anisotropy barrier, E_H_ is the Zeeman energy, and E_D_ is the dipolar interaction energy. As a consequence, it is more convenient to picture the effect of dipolar interaction energy (E_D_) to analyze the improvement of heat dissipation of the surface encapsulated nanoparticles (although the effects of E_H_ can be negligible as followed by the Hergt-Dutz limit^74^). The assumption is justified considering the dipolar energy formula^75^, E_D_ = αμ^2^/${d}_{ij}^{3}$, where α is a constant, μ represents the magnetic strength of the MNPs, and d is the separation between two particles *i* and *j*. Therefore, E_D_ imparts a disordering torque, disarranging the spin relaxation mechanism owing to the competition between intraparticle interactions and anisotropy among the nanoparticles^76^. This competition leads to frustrated magnetic moments or disordered easy anisotropy axis orientations, resulting in a demagnetizing effect within the system^77^. This effect could possibly lead to decreased heating efficiency in uncoated MNPs. Another noteworthy finding from Fig. 10 is that the heating response is more pronounced in the CEuFO case than in the DEuFO system. As evident from the TEM analysis, CEuFO MNPs exhibit greater dispersion than DEuFO MNPs, leading to reduced dipolar interaction energy that enhances the SAR. Furthermore, Fig. 11 depicts a range of alignments for a pair of magnetic dipoles, ordered from the lowest to the highest dipole energy configurations. In the initial strategy presented in Fig. 11, the dipole moments are aligned along the vector r in a linear chain configuration, all possessing the same polarity. This arrangement represents the most stable orientation. The tendency to form such chains has significant implications for clustering MNPs and their efficiency in heat generation. Conversely, an antiparallel alignment becomes the most favorable when the dipole moments are oriented perpendicular to the vector r. In our study, mutual dipole interactions can be successfully control via appropriate coating of dextran and chitosan, which tends to increase the separation between the magnetic dipoles, which further improved the self-heating efficiency. Based on this, the chitosan-coated Eu-doped Fe_3_O_4_ MNPs portray a rising heating efficiency among all the fabricated samples, rendering them a safe and efficient agent for hyperthermia applications.

## Conclusion

In summary, this study outlines a method for designing Eu-doped FO nanoparticles functionalized with chitosan and dextran, representing how biopolymer encapsulation offers a promising approach to control agglomeration of the reference MNPs. The investigation delves into the impact of controlling dipolar interactions through the relaxation mechanism. The inclusion of chitosan and dextran in the EuFO MNPs contributes to precise structural control and fine-tuned magnetic properties, further enhancing the self-heating efficiency of the characterized MNPs. In particular, the results indicate that the reported SAR value of 762.21 W/g for CEuFO MNPs, which is under safety limit, could open avenues for future investigations to explore the potential use of these MNPs in vivo and optimize clinical parameters, prefiguring an intriguing new path toward successful MFH applications.

## Data Availability

The data that support the findings of this study are available within the article.
